# KCNQ1-deficient and KCNQ1-mutant human embryonic stem cell-derived cardiomyocytes for modeling QT prolongation

**DOI:** 10.1186/s13287-022-02964-3

**Published:** 2022-06-28

**Authors:** Yuanxiu Song, Tianwei Guo, Youxu Jiang, Min Zhu, Hongyue Wang, Wenjing Lu, Mengqi Jiang, Man Qi, Feng Lan, Ming Cui

**Affiliations:** 1 Department of Cardiology, Peking University Third Hospital, 49 Huayuan North Road, Haidian District, Beijing, 100191 China; 2 Beijing Lab for Cardiovascular Precision Medicine, Anzhen Hospital, Capital Medical University, Beijing, 100029 China; 3 State Key Laboratory of Cardiovascular Disease, Fuwai Hospital, National Center for Cardiovascular Diseases, Chinese Academy of Medical Sciences and Peking Union Medical College, Beijing, 100037 China; 4 Shenzhen Key Laboratory of Cardiovascular Disease, Fuwai Hospital Chinese Academy of Medical Sciences, Chinese Academy of Medical Sciences and Peking Union Medical College, Shenzhen, 518057 China; 5 Department of Cell Biology, School of Basic Medical Sciences, Peking University Health Science Center, Beijing, 100191 China; 6 Department of Cardiology, The Second Affiliated Hospital of Zhengzhou University, Jingba Road, Zhengzhou, 450053 China

**Keywords:** Iks, KCNQ1, LQT, hESCs, CRISPR/cas9

## Abstract

**Background:**

The slowly activated delayed rectifier potassium current (I_Ks_) mediated by the KCNQ1 gene is one of the main currents involved in repolarization. *KCNQ1* mutation can result in long-QT syndrome type 1 (LQT1). I_Ks_ does not participate in repolarization in mice; thus, no good model is currently available for research on the mechanism of and drug screening for LQT1. In this study, we established a *KCNQ1*-deficient human cardiomyocyte (CM) model and performed a series of microelectrode array (MEA) detection experiments on *KCNQ1*-mutant CMs constructed in other studies to explore the pathogenic mechanism of *KCNQ1* deletion and mutation and perform drug screening.

**Method:**

KCNQ1 was knocked out in human embryonic stem cell (hESC) H9 line using the CRISPR/cas9 system. *KCNQ1*-deficient and *KCNQ1*-mutant hESCs were differentiated into CMs through a chemically defined differentiation protocol. Subsequently, high-throughput MEA analysis and drug intervention were performed to determine the electrophysiological characteristics of *KCNQ1*-deficient and *KCNQ1*-mutant CMs.

**Results:**

During high-throughput MEA analysis, the electric field potential and action potential durations in *KCNQ1*-deficient CMs were significantly longer than those in wild-type CMs. *KCNQ1*-deficient CMs also showed an irregular rhythm. Furthermore, *KCNQ1*-deficient and *KCNQ1*-mutant CMs showed different responses to different drug treatments, which reflected the differences in their pathogenic mechanisms.

**Conclusion:**

We established a human CM model with *KCNQ1* deficiency showing a prolonged QT interval and an irregular heart rhythm. Further, we used various drugs to treat *KCNQ1*-deficient and *KCNQ1*-mutant CMs, and the three models showed different responses to these drugs. These models can be used as important tools for studying the different pathogenic mechanisms of *KCNQ1* mutation and the relationship between the genotype and phenotype of *KCNQ1*, thereby facilitating drug development.

**Supplementary Information:**

The online version contains supplementary material available at 10.1186/s13287-022-02964-3.

## Introduction

Long-QT syndrome (LQTS) is a cardiogenetic disorder that can cause life-threatening arrhythmias and is associated with sudden cardiac death [1, 2]. Long-QT syndrome type 1 (LQT1) is the most prevalent subtype, accounting for approximately 40–50% of all patients with LQTS [3]. Studies have reported that LQT1 is caused by loss-of-function mutations in *KCNQ1* that encodes the α subunit of the cardiac Kv7.1 potassium channel mediating the slowly activated delayed rectifier potassium current (I_Ks_) [4–9]. To date, numerous mutations in *KCNQ1* have been considered responsible for hereditary LQT1, and the type and location of *KCNQ1* mutation are associated with varying clinical severities [10–13]. However, the clinical phenotype of the different mutations and the underlying mechanisms remain poorly understood.

Studies have demonstrated that mutations at the N-terminus of *KCNQ1*, such as Y111C, L114P, and P117L, could affect it transport [14]. Moreover, mutations on the C-loop inactivate the I_Ks_ mediated by protein kinase A (PKA), causing the inward calcium ion current to increase without confrontation during β-adrenergic stimulation [8]. These studies have provided direct evidence demonstrating that the types of mutation are differently associated with physiological activities and mechanisms; this provides the theoretical basis for drug target screening. However, most experiments have been performed almost exclusively on animal models [15, 16]. Although *KCNQ1*-knockout mice presented with the Jervell and Lange–Nielsen phenotype [17, 18], the mouse heart repolarization K+ current is a fast, slow-transient outward current, and delayed rectified voltage-gated K^+^ current, which cannot reflect the effect of *KCNQ1* deficiency in humans [19]. Hence, the establishment of *KCNQ1*-mutant human embryonic stem cell-derived cardiomyocytes (CMs) is necessary for the mechanistic exploration and drug screening for LQT1 caused by *KCNQ1* mutations.

In recent years, the establishment of disease models and drug screening using human induced pluripotent stem cell-derived CMs (hiPSC-CMs) has become a promising therapeutic approach for cardiovascular diseases [20]. In this study, we developed *KCNQ1*-deficient, KCNQ1^L114P/+^, and KCNQ1^R190Q/+^ human myocardial models using CRISPR/Cas9 system to investigate a well-defined genotype–phenotype correspondence. *KCNQ1*-deficient cells showed serious QT prolongation, irregular rhythm, early post-depolarization (EAD), and I_Kr_ current insensitivity. KCNQ1^L114P/+^ CMs showed a significantly longer QT delay than KCNQ1^R190Q/+^ CMs. Our results showed that MgCl2, propranolol, and amiodarone could reverse the abnormal phenotype caused by *KCNQ1* deficiency or mutations separately. The results showed that those models can well reflect the disease phenotype and contribute to the drug screening and accurate treatment of *KCNQ1* mutation-related diseases.

## Methods

### Cell culture and cardiac differentiation of hESC

The hESC line was purchased from Cellapy (Beijing, China) was routinely maintained in the presence of PSCeasy medium (Cellapy, China) on six-well plates (Corning, USA) coated with 5% Matrigel (Corning, USA). Medium was changed every day and passaged every 2–3 days with EDTA (Cellapy, China). The cells were grown in a humidified atmosphere of 95% air and 5% CO_2_ at 37 °C. The hESCs were differentiated when they reached 70–80% confluence. Medium was changed to the basal differentiation medium. For day 0 to day 2, medium was changed to the basal medium C01 (Cellapy, China). For day 2 to day 4, medium was changed to the basal differentiational medium C02 (Cellapy, China). On day 4, medium was changed to the basal differentiational medium C03 (Cellapy, China) and changed medium every other day. Contracting cells were noted from day 9.

### Genome editing

KCNQ1 single-stranded guide RNA (sgRNA) (ATGCTACACGTCGACCGCCA,CAGCCGCCCCCAGAGGCCCA,GCTCGAGGAAGTTGTAGACG) was designed using an online tool (https://www.synthego.com). We electroporated the epiCRISPR vector and sgRNA (100 µl electrotransformation solution (Cellapy, China) plus 2.5 µg KCNQ2 gRNA plasmid) into the cells using the 4D nuclear receptor system and the CA137 programme (Lonza, Germany). The transfected cells were seeded in 6-well plates and cultured overnight in PSCeasy medium 10 μM of Rho kinase inhibitor Y-27632. The medium was changed the next day. Drug (puromycin) selection was initiated after 72 h of transfection at a lower concentration of 0.1 µg/ml for the first hour and then at 0.3 µg/ml until the transfected lines were stable. The surviving cells were collected in 48-well plates and amplified for polymerase chain reaction (PCR) screening. The point mutation cells were prepared by epi-ABEmax/epi-AncBE4max/epi-ABEmax-NG/epi-AncBE4 max-NG plasmid. The plasmid was transfected using Lipofectamine 3000, and then, the transfected cells were selected by blasticidin. The specific methods can refer to the previous research.

### Drug treatment

100 mM 293B, 100 µM isoproterenol (ISO), 5 µM propranolol, 100 µM amiodarone 100 µM MgCl_2_ (Selleck, USA) were diluted in C05 (Cellapy, China). hESC-CMs were treated with 293B, ISO, Propranolol for 12 h. hESC-CMs were treated with Amiodarone, MgCl_2_ for 30 min.

### RNA extraction and RT-PCR

Total RNA from cells was extracted by using TRIZOL Reagent (Invitrogen, USA). An amount of 2 µg total RNA was reversed to cDNA by using the GoScript Reverse Transcription System (Promega, USA). Quantitative RT-PCR involved use of SYBR Green II (Takara, Japan) in the iQ5 system (Bio Rad, Hercules, CA). A comparative CT method was used to analyze the relative changes in gene expression. The results were expressed as relative to the data of GAPDH transcripts (internal control). Primer sequences are listed in Additional file 1: Table S1.

### Immunofluorescent staining (IF) and imaging analyses

The cells were plated on 20 mm coverslips coated with 5% Matrigel and were fixed with 4% PFA for 15 min. Then, after washing with PBS three times for 5 min, the cells were permeabilized with 0.2% Triton X-100 (Sigma, USA) for 15 min and blocked with 3% BSA (Sigma, USA) for 1 h at room temperature. After that cells were incubated with primary antibodies, overnight at 4 °C. Then, cells were washed by PBS and incubated for 1 h at room temperature in the dark with secondary antibodies (Invitrogen, USA). Cells were washed again as above, mounted with Fluoroshield Mounting Medium with DAPI (4, 6 diamino-2-phenylindole). Images were taken under a Confocal Microscope (Leica DMI 4000B, German). The antibody and their appropriate dilution are provided in Additional file 1: Table S2.

### Western blot (WB) analysis

Protein from hESC-CMs was extracted by using a Protein Extraction Kit (Promega, USA). The protein concentration of the supernatant was measured by BCA method. The 30 µg protein was separated on 10% SDS-PAGE and transferred to PVDF membrane at 300 mA for 90 min, which was blocked with 5% albumin bovine (BSA) at room temperature for 1 h, then incubated at 4 °C overnight with the primary antibodies, then with IR dye-conjugated secondary antibodies (LI-COR, USA) for 1 h at room temperature. GAPDH was used as an internal control. Blots were exposed and analyzed with use of an Odyssey infrared imaging system (LI-COR Biosciences, USA). The antibody and their appropriate dilution are provided in Additional file 1: Table S2.

### Flow cytometry

The hESC-CMs under different treatments were singularized with CardioEasy Human Cardiomyocyte Digestive Fluid (Cellapy, China). Observe that most of the clones are detached from the bottom of the plate under the microscope, gently pipette the cells and suck them out, centrifuge, and wash three times with PBS. The cells were stained with different antibodies, filtered through the 300 mesh filter, and immediately analyzed by FACS (Beckman, USA). The cell count is generally 1–2 million. The results were analyzed with Flow Jo X program.

### Microelectrode array (MEA) analysis

hESC-CMs were digested in CardioEasy Human Cardiomyocyte Digestive Fluid (Cellapy, China), after which 2 × 104 cells were plated on a microelectrode array (MEA) pre-coated with 5% Matrigel (Cellapy, China). The next day, 300 μl medium was added to each well. After the hESC-CMs resumed spontaneous beating, the experimental data were recorded on a Maestro EDGE (Axion Biosystems, Inc., Atlanta, USA) according to the MEA manual. Cardiac Analysis Tool, AxIS Navigator, AxIS data export tool, and Origin were used to analyze the data.

### Statistical analysis

Results are expressed as mean ± SD. Statistical analysis was performed with GraphPad Prism 8.00 for Windows. Two-sided unpaired Student’s *t* test was used to compare 2 groups with normal distribution. One-way ANOVA was used to compare 3 or more groups. All tests for normality and homogeneity of variance were passed before t test and one-way analysis of variance. *P* values of less than 0.05 were used to denote statistical significance. *P < 0.05; **P < 0.01, ***P < 0.001; NS, not significant.

## Results

### Establishment of the *KCNQ1*-knockout and *KCNQ1*-mutant hESC-CM models

**Fig. 1:**
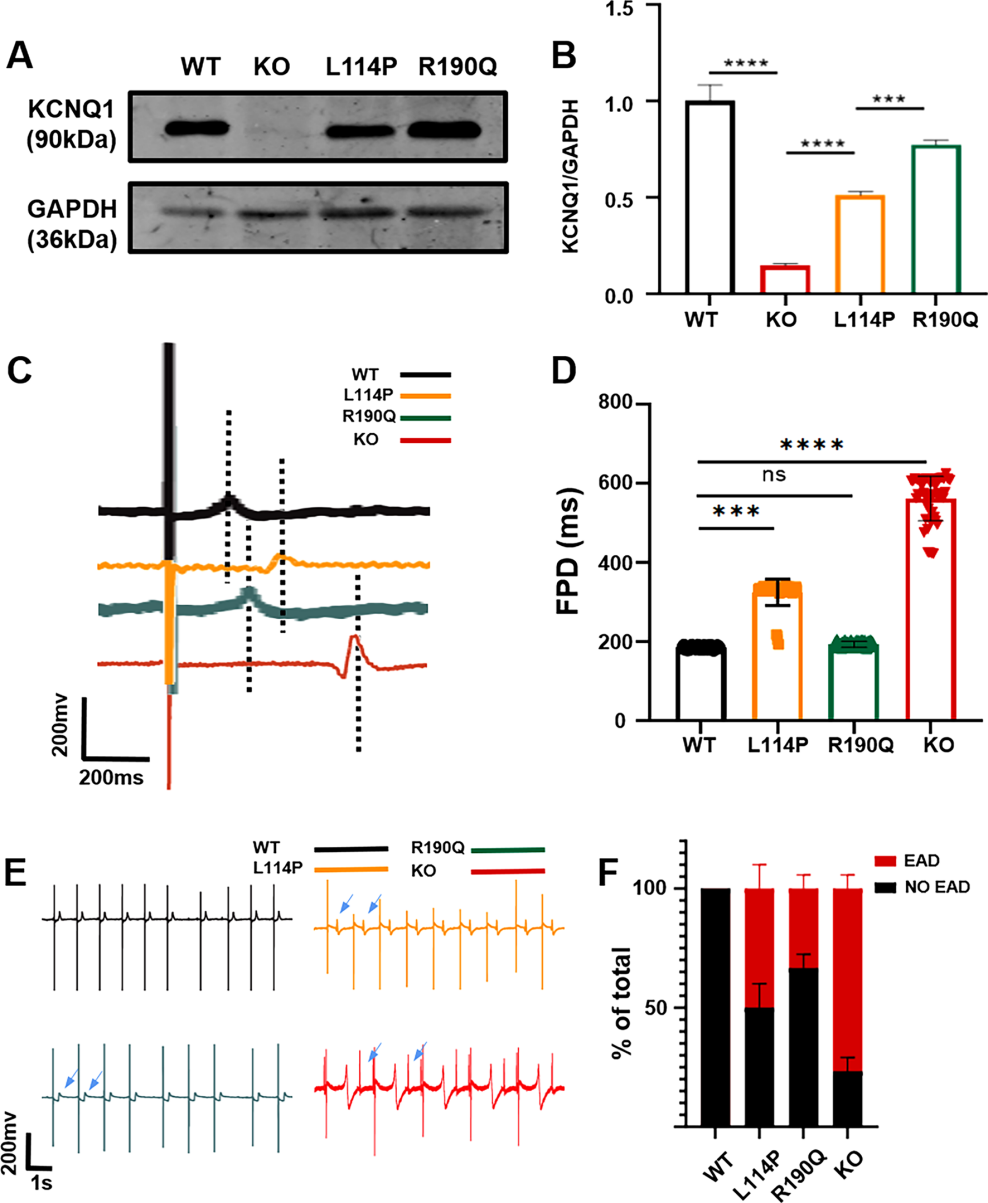
The baseline of four CMs. **A**,** B** Western blotting showed the expression of KCNQ1 in four hESC cells and quantitative analysis. **C** MEA recorded representative FPD waveforms on four CMs. **D** Representative FPD signal and quantitative analysis of four CMs. **E** MEA recorded representative rhythm traces on four CMs. **F** MEA quantitative analysis showed the proportion of EAD in four CMs. Results are presented as means ± standard error of the mean (n = 3, *P < 0.05, **P < 0.01, ***P < 0.001, ****P < 0.0001)

We used the CRISPR/Cas9 system to establish a *KCNQ1*-deficient H9 hESC cell model. We designed a highly specific sgRNA targeting KCNQ1 and electroporated hESC H9 cells with a plasmid containing sgRNA and Cas9 element. Subsequently, the transfected cells were screened using puromycin, and the genotype of the surviving clones was determined using the Sanger sequencing method. Sequencing results revealed that a homozygous clone with a 2-basepair(bp) mutation in *KCNQ1* was obtained (KCNQ1^R190Q/+^) (Additional file 2: Fig. S1A). The pluripotency of KCNQ1^R190Q/+^ was identified using the appropriate markers, and the karyotype and tumorigenic characteristics of stem cells did not change (Fig. S1B–E) KCNQ1^R190Q/+^ and other hESCs (KCNQ1^L114P/+^ and KCNQ1^−/−^) that were established in our previous work were induced to differentiate into CMs using small molecules with clear chemical compositions (Additional file 3: Fig. S2A) [21]. Immunofluorescence staining of CMs for 30 days showed normal expression of troponin T (TNNT2) and α-actin (Additional file 3: Fig. S2B). Flow cytometry revealed that the TNNT2 positivity rate of H9 hESC wild type (WT) and KCNQ1^−/−^ CMs was close to 86% (Additional file 3: Fig. S2C and S2D). We tested the cell viability of the four kinds of hESC-CMs by CCK8, and the results showed that there was no difference in the cell viability of the four kinds of hESC-CMs (Additional file 3: Fig. S2E). Western blot analysis confirmed the absence of the KCNQ1 protein in KCNQ1^−/−^ CMs (Fig. 1A, B, Additional file 3: Fig. S2F). Furthermore, the expression of *KCNQ1* in KCNQ1^L114P/+^ CMs significantly decreased compared with that in WT and KCNQ1^R190Q/+^ CMs, suggesting the abnormal KCNQ1 transport of KCNQ1^L114P/+^ CMs.

### KCNQ1^−/−^, KCNQ1^L114P/+^, and KCNQ1^R190Q/+^ CM models reflect the LQT phenotype

Differences between KCNQ1^−/−^, KCNQ1^L114P/+^, and KCNQ1^R190Q/+^ CMs were observed at the multicellular level using the high-throughput Maestro Edge microelectrode array (MEA) system [22, 23]. The field potential duration (FPD) was calculated as the time between depolarization and repolarization marked by the beat time and the repolarization peak or T-wave, respectively. FPD can reflect the duration of myocardial QT interval. FPD statistics showed no difference between KCNQ1^R190Q/+^ and WT CMs. Moreover, the FPD of KCNQ1^L114P/+^ CMs was only slightly prolonged, whereas that of KCNQ1^−/−^ CMs was significantly prolonged (Fig. 1C, D). This slight prolongation in KCNQ1^L114P/+^ CMs may be due to the lack of repolarization I_Ks_ caused by abnormal KCNQ1 transport, which is consistent with the decreased expression of *KCNQ1* in KCNQ1^L114P/+^ CMs as observed through Western blot analysis. Irregular rhythm and EADs are precursors of ventricular arrhythmias in LQT; therefore, we also used MEA to analyze the rhythm of the three models. The results indicated that KCNQ1^−/−^ CMs show obvious arrhythmia and that the proportion of EADs significantly increased compared with WT CMs. KCNQ1^L114P/+^ and KCNQ1^R190Q/+^ CMs also showed obvious arrhythmia (Fig. 1E, F).

### Response to I_Ks_-specific blocker

**Fig. 2:**
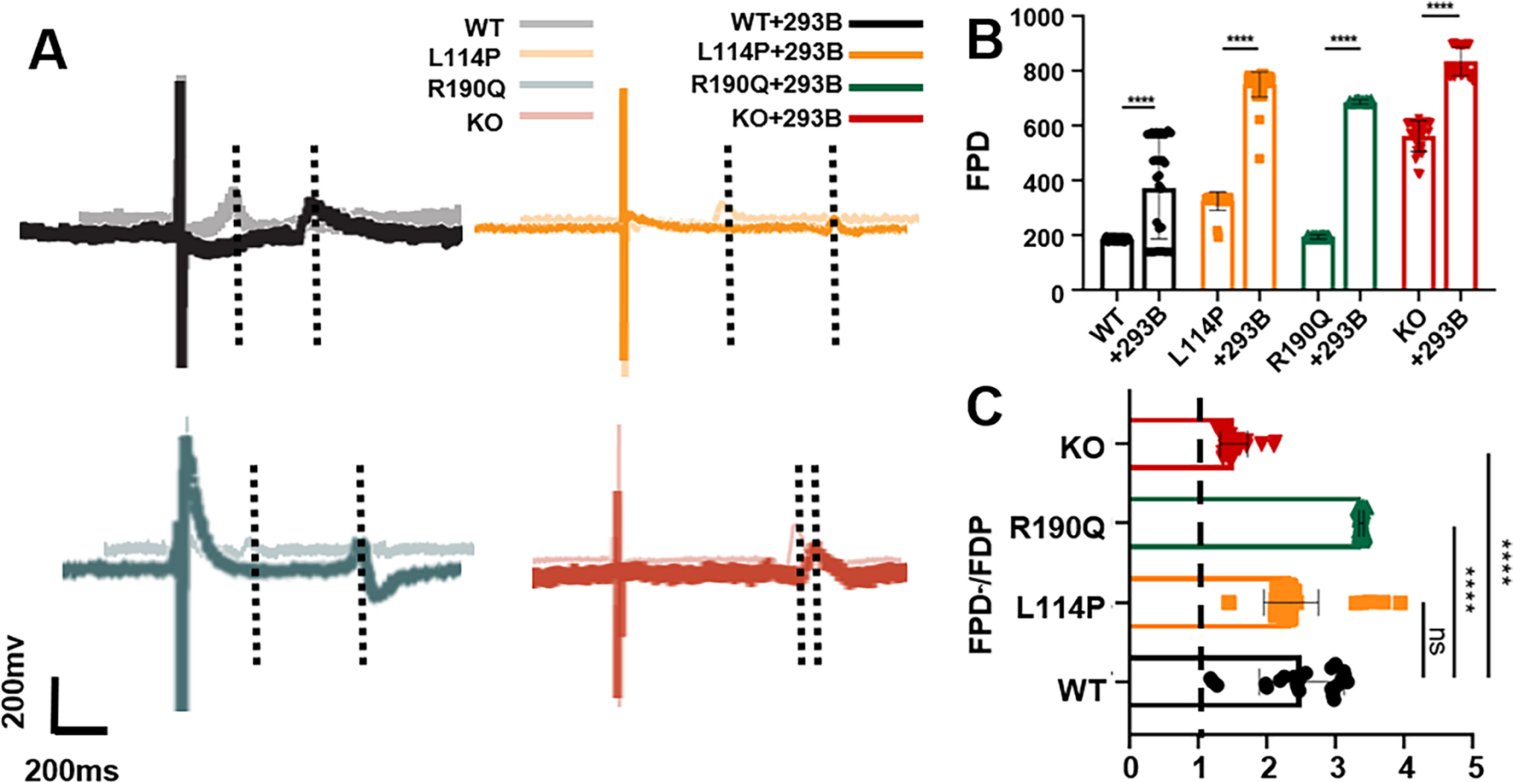
Four CMs in response to 293B. **A**, **B** Representative FPD and quantitative analysis of four CMs after treatment with 100-mΜ 293B. **C** Ratio of the FDP to baseline of four CMs after 293B treatment. Results are presented as means ± standard error of the mean (n = 3, *P < 0.05, **P < 0.01, ***P < 0.001, ****P < 0.0001)

Moreover, we tested the effects of the I_Ks_-specific blocker chromanol 293B on the FPD of the three models [24]. We used the ratio of FPD after dosing to that at baseline (FDP'/FDP) to indicate the extent of FPD change. A value of > 1 indicates that the FPD is prolonged, whereas a value of < 1 indicates a shortened FPD. Interestingly, the FPD of WT, KCNQ1^L114P/+^, and KCNQ1^R190Q/+^ CMs showed prolongation after treatment with 100-mM 293B. In contrast, the FPD of KCNQ1^−/−^ CMs did not show significant prolongation as that of KCNQ1^L114P/+^ and KCNQ1^R190Q/+^ CMs after treatment with 293B (Fig. 2A, B). Meanwhile, the FDP'/FDP of WT, KCNQ1^L114P/+^, and KCNQ1^R190Q/+^ CMs was significantly higher than that of KCNQ1^−/−^ CMs; however, the FPD prolongation of the knockout CMs is the least obvious (Fig. 2C). These results indicate that the *KCNQ1* mutation and knockout models were successfully established.

### Responses to MgCl_2_

**Fig. 3:**
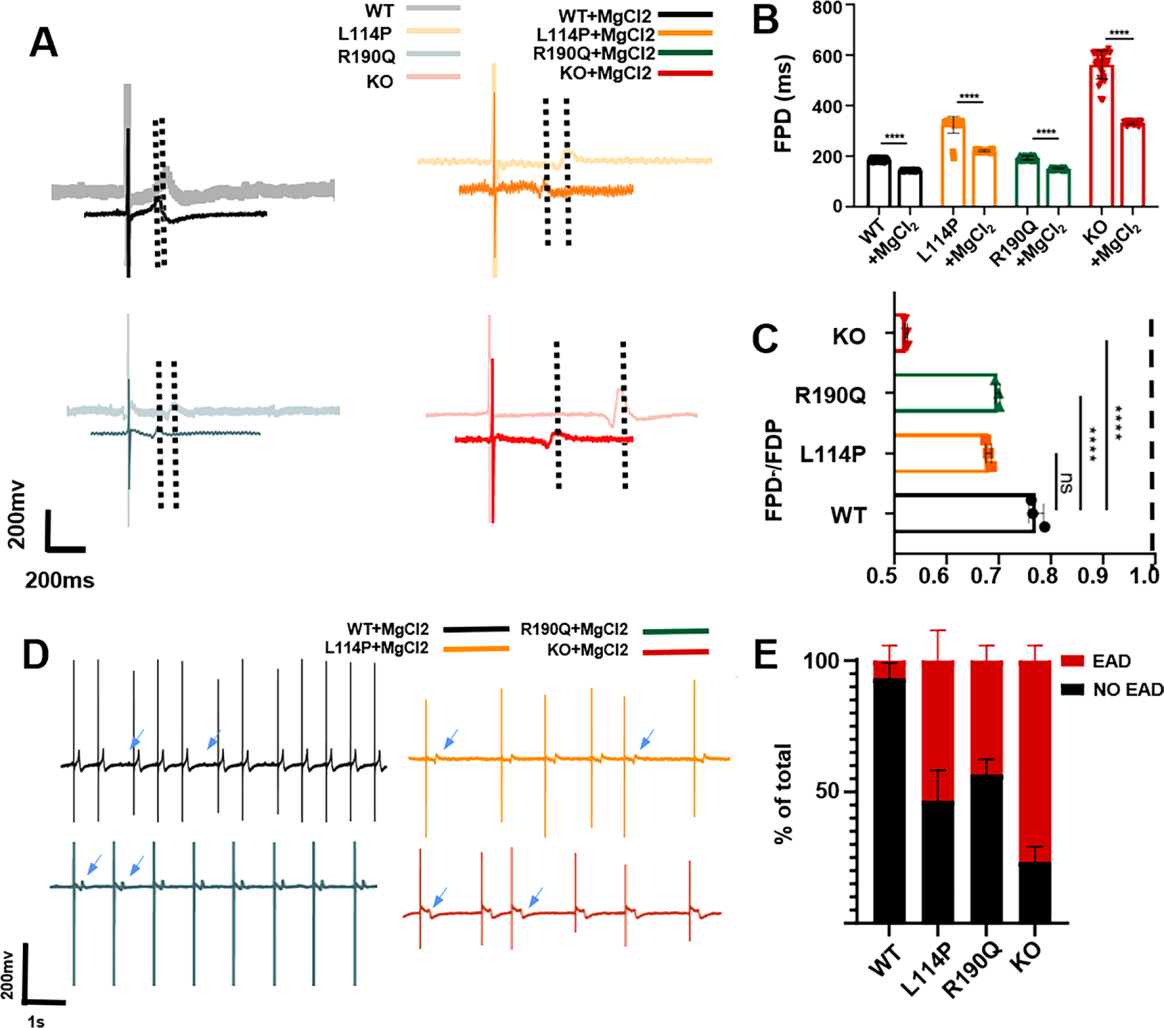
Four CMs in response to MgCl_2_. **A**, **B** Representative FPD and quantitative analysis of four CMs after treatment with 1-mM MgCl_2_. **C** Ratio of the FDP to baseline of four CMs after MgCl_2_ treatment. **D** Representative traces of EAD in the FPD of four CMs after treatment with MgCl_2_. **E** Quantitative analysis showed the proportion of EAD in four CMs after treatment with MgCl_2_. Results are presented as means ± standard error of the mean (n = 3, *P < 0.05, **P < 0.01, ***P < 0.001, ****P < 0.0001)

Mg^2+^ is the main coenzyme for potassium ion transfer inside and outside a cell. Mg^2+^ supplementation can increase potassium ion transport, increase the intracellular potassium concentration, and increase stability of cell membrane and electrocardiogram. Therefore, we observed changes in the FPD of the four CMs after MgCl_2_ treatment. MgCl_2_ treatment can shorten the FPD of all three models, with the FPD shortening of KCNQ1^−/−^ CMs being the most significant (Fig. 3A–C). This suggests that magnesium supplementation is essential for LQT treatment with different mechanisms. However, EAD cannot be eliminated (Fig. 3D, E).

### Responses to isoproterenol

**Fig. 4:**
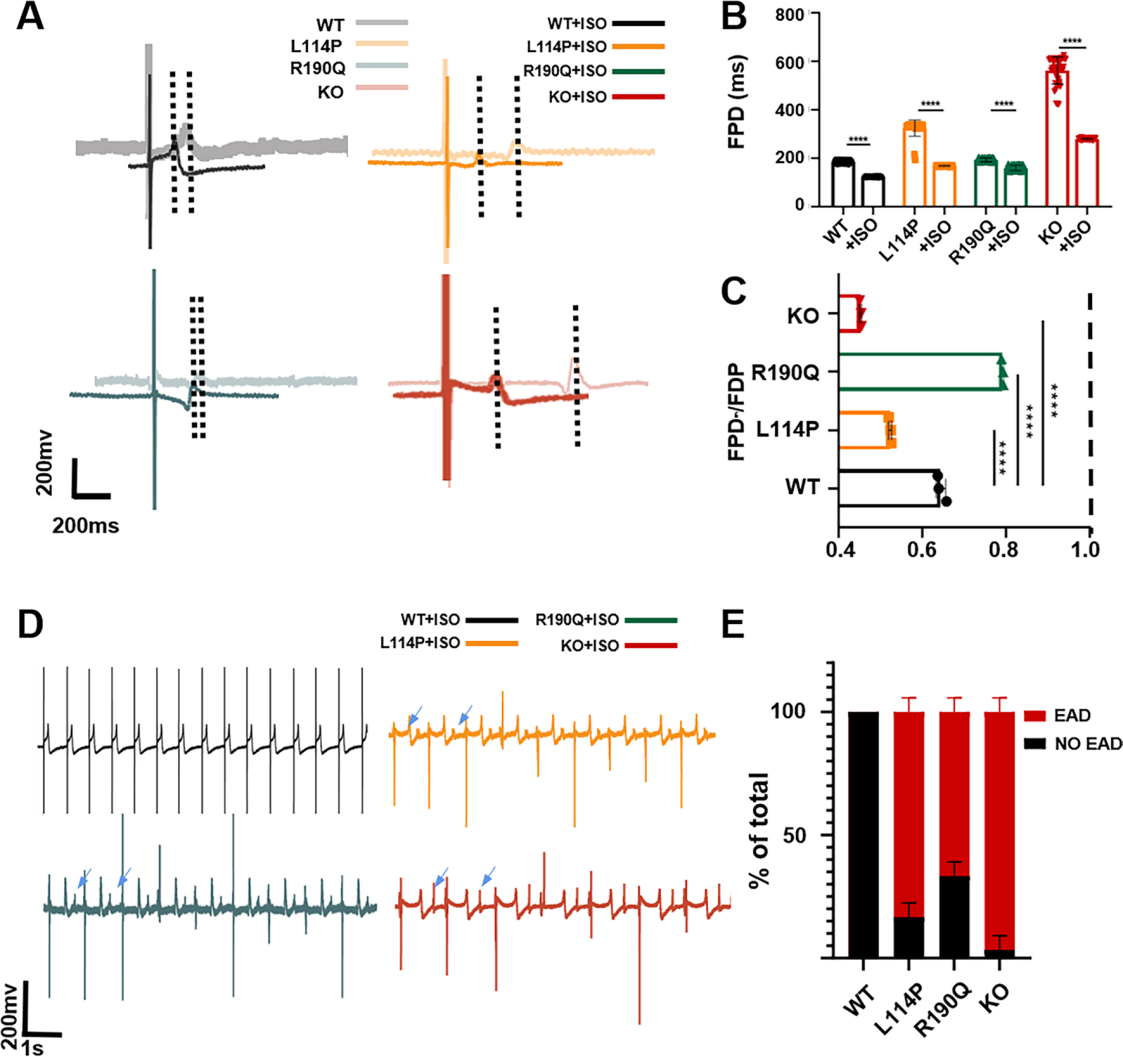
Four CMs in response to ISO. **A**, **B** Representative FPD and quantitative analysis of four CMs after treatment with 100-uM ISO. **C** Ratio of the FDP to baseline of four CMs after ISO treatment. **D** Representative traces of EAD in the FPD of four CMs after treatment with ISO. **E** Quantitative analysis showed the proportion of EAD in four CMs after treatment with ISO. Results are presented as means ± standard error of the mean (n = 3, *P < 0.05, **P < 0.01, ***P < 0.001, ****P < 0.0001)

The occurrence of LQT1 is often associated with sympathetic nerve excitement (such as due to exercise or emotional agitation); therefore, sympathetic nerve excitement was simulated using isoproterenol (ISO) treatment [25]. ISO is a β-agonist that binds to β-AR and activates cAMP–PKA-dependent downstream signals, which then promotes the phosphorylation of several target proteins, including the L-type calcium channel and lysine receptor. Subsequently, PKA phosphorylates these proteins and increases the calcium concentration in the sarcoplasm, resulting in the activation of the crossbridge and further enhancement of the contraction of CMs [26]. After treatment with 100-μM ISO, the FPD of WT, KCNQ1^L114P/+^, and KCNQ1^−/−^ CMs was significantly shortened compared with the baseline (Fig. 4A–C). This phenotypic change was caused by the agonistic effect of ISO. However, the FPD of KCNQ1^R190Q/+^ CMs did not appear to be significantly shortened, suggesting that KCNQ1^R190Q/+^ CMs are not sensitive to ISO. This is consistent with the results of previous studies demonstrating that mutations in the C-loop, such as those in KCNQ1^R190Q/+^ CMs, may indeed inactivate Kv7.1’s response to PKA stimulation [12]. Furthermore, WT CMs did not show arrhythmia after ISO treatment, whereas KCNQ1^L114P/+^ CMs and KCNQ1^R190Q/+^ CMs both showed arrhythmia aggravation (Fig. 4D, E). Both point mutation cells showed the arrhythmia phenotype under β-adrenergic stimulation, which was consistent with the phenotype where LQT1 was more easily induced under sympathetic excitation; this indicates that the point mutation model can reflect the response of the myocardium to sympathetic excitation.

### Responses to propranolol

**Fig. 5:**
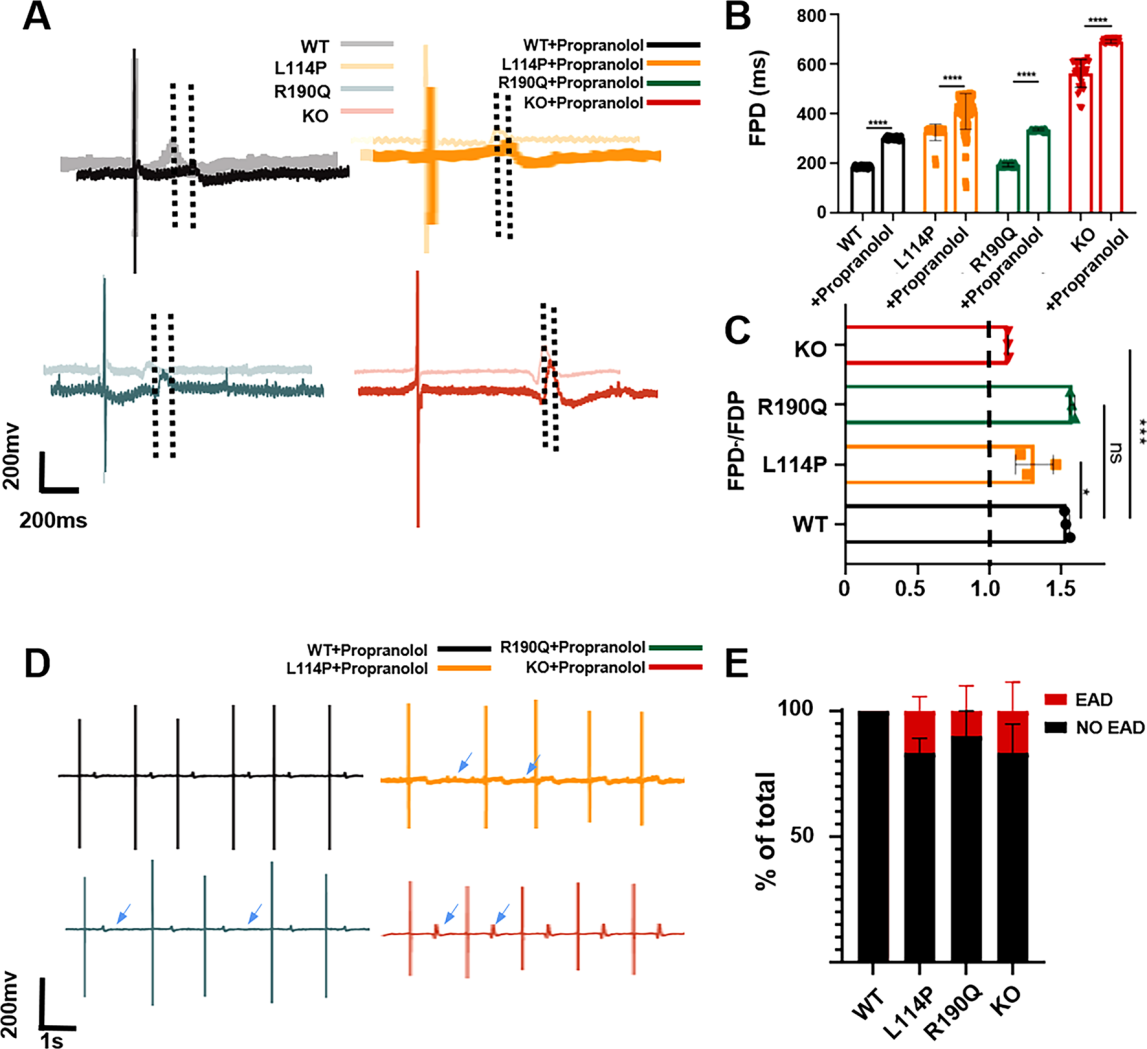
Four CMs in response to propranolol. **A**, **B** Representative FPD and quantitative analysis of four CMs after treatment with 5-uM propranolol. **C** Ratio of the FDP to baseline of four CMs after propranolol treatment. **D** Representative traces of EAD in the FPD of four CMs after treatment with propranolol. **E** Quantitative analysis showed the proportion of EAD in four CMs after treatment with propranolol. Results are presented as means ± standard error of the mean (n = 3, *P < 0.05, **P < 0.01, ***P < 0.001, ****P < 0.0001)

β-blockers can directly decrease β-adrenergic signaling and show antiarrhythmic effects [26]. Propranolol, a β-blocker, is one of the most common drugs used in the clinical treatment of LQT. Therefore, we subjected the CM models to this drug and examined the responses. The results showed that the three CM models showed prolongation of the FPD (Fig. 5A–C). Moreover, the slowing of the heart rhythm and arrhythmia in KCNQ1^−/−^, KCNQ1^L114P/+^, and KCNQ1^R190Q/+^ CMs was significantly decreased compared with the baseline values (Fig. 5D, E). Propranolol can improve the arrhythmia phenotype of the three *KCNQ1*-mutant myocardial models, including KCNQ1^−/−^ CMs, indicating that propranolol has therapeutic effects on LQT1 through multiple mechanisms**.**

### Responses to amiodarone

**Fig. 6:**
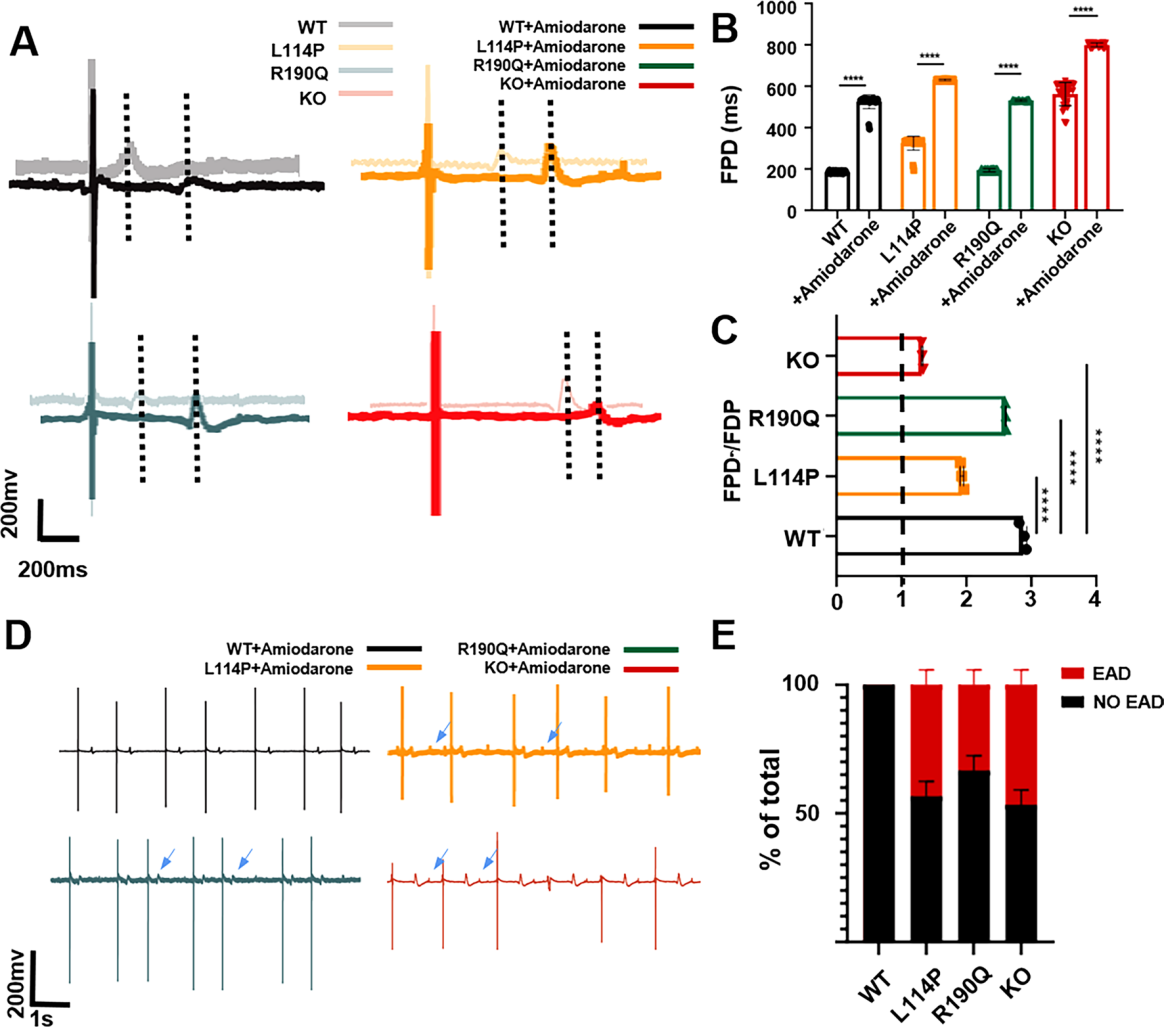
Four CMs in response to Amiodarone. **A**, **B** Representative FPD and quantitative analysis of four CMs after treatment with 100-uM amiodarone. **C** Ratio of the FDP to baseline of four CMs after amiodarone treatment. **D** Representative traces of EAD in the FPD of four CMs after treatment with amiodarone. **E** Quantitative analysis showed the proportion of EAD in four CMs after treatment with amiodarone. Results are presented as means ± standard error of the mean (n = 3, *P < 0.05, **P < 0.01, ***P < 0.001, ****P < 0.0001)

We also tested the response of the three models to other common LQT medications used clinically. Amiodarone is a type III multi-ion channel blocker that can selectively prolong the repolarization time of the myocardium and is suitable for various ventricular arrhythmias [27]. After treatment with 100-μM amiodarone, WT, KCNQ1^L114P/+^, KCNQ1^R190Q/+^, and KCNQ1^−/−^ CMs showed significant FPD prolongation; however, KCNQ1^−/−^ CMs had the smallest FPD extension (Fig. 6A–C). Amiodarone alleviated the arrhythmia phenotype of KCNQ1^L114P/+^ CMs but weakened the pulsation of KCNQ1^−/−^ CMs (Fig. 6D, E). These results suggest that amiodarone has a good therapeutic effect on LQT1 with different mutations; however, it is not suitable for treating patients with *KCNQ1* large fragment deletion.

## Discussion

*KCNQ1* mutation is strongly correlated with LQT1. In this study, we used MEA to investigate the response of different genotypes of *KCNQ1* mutations to different drugs to determine the underlying mechanisms of different *KCNQ1* mutations. Our results indicated that *KCNQ1*-deficient and *KCNQ1*-mutant cells showed varying serious QT prolongation and irregular rhythm and that these can be corrected using I_Ks_-specific, β, and multi-ion channel blockers. These results suggest that the novel hiPSC-CM models are helpful tools in the determination of pathogenic mechanisms and drug screening for LQT1 induced by *KCNQ1* mutations.

Congenital LQTS is a life-threatening arrhythmic syndrome and is the leading cause of sudden death among young people [28]. The typical characteristics of LQTS are prolongation of the QT interval on electrocardiography and the presence of syncope or cardiac arrest mainly due to emotional or physical stress. The three main genotypes of LQTS—LQT1, LQT2, and LQT3—account for 80–90% of all 15 gene mutations found in patients with LQTS [14]. As the main genetic genotype of LQT, LQT1 is caused by mutations in the slow potassium (K+ outward current channel encoded by *KCNQ1* [29]). *KCNQ1* encodes the α-subunit of the K+ channel Kv7.1, producing a depolarized I_Ks_ current that increases through sympathetic activation and is critical for QT adaptation as the heart rate increases [30]. When I_Ks_ is defective, the QT interval cannot be appropriately shortened during tachycardia, resulting in high-grade arrhythmia. Homozygous mutations or compound heterozygous mutations in *KCNQ1* can lead to Jervell and Lange–Nielsen syndrome, which is characterized by decreased inner ear I_Ks_ and deafness [31]. There are more than 100 pathogenic heterozygous mutations in *KCNQ1*, each with a different effect on the polymeric K+ channel. Mutant and WT protein subunits may assemble together and have a significant negative effect on the current. Alternatively, certain mutant subunits may fail to co-assemble with the WT peptide, resulting in the loss of function that reduces I_Ks_ by ≤ 50% (haploinsufficiency). The latter may also be due to mutations interfering with intracellular subunit transport, preventing the mutant peptide from reaching the cell membrane.

The complex mutation mechanism increases the difficulty of providing precise treatment for LQT1; therefore, establishing an effective model is essential to enable mechanism exploration and drug screening. Due to the huge difference in the cardiac functions of mice and humans, I_Ks_ does not participate in repolarization in mice, making it difficult to use mice models. HiPSC-CMs can simulate human cardiac action potential, which is a good application prospect in disease modeling. Models constructed using patient-derived hiPSC-CMs have been shown to be effective [32]. However, patient-derived hiPSC-CMs cannot accurately reflect the gene–phenotype relationship due to the influence of background genes. Moreover, obtaining patient-derived cardiac muscles is difficult. Therefore, the use of gene editing to artificially prepare myocardium with point mutation for disease simulation has good application prospects. In previous studies, our research group constructed KCNQ1^L114P/+^ and KCNQ1^R190Q/+^ models using BaseEditor’s method.

KCNQ1^L114P/+^ and KCNQ1^R190Q/+^ are serious pathogenic mutations in the LQT1 phenotypes. The pathogenic mechanism of LQT1 caused by KCNQ1 mutation mainly includes the following categories: defects in ion permeation, channel gating, trafficking defect, KCNQ1–KCNE1 interaction, PKA-mediated signaling pathway, PIP2 binding, and calmodulin binding [33]. KCNQ1^L114P/+^ is a mutation at the N-terminus of *KCNQ1* that affects the upper membrane transport of *KCNQ1* [21], whereas KCNQ1^R190Q/+^ is a mutation located on the C-ring of *KCNQ1*. Studies have proven that such mutations may reduce the sensitivity of *KCNQ1* to PKA, leading to the inability of I_Ks_ to significantly increase when stimulated by adrenaline, thus inducing arrhythmias. The electrophysiological phenotypes of the two models were preliminarily detected and found to be able to reflect the disease phenotypes. Furthermore, due to the diverse pathogenic mechanisms of *KCNQ1*, there is no effective human *KCNQ1* deletion model to clarify the direct phenotype of *KCNQ1*. Large fragment *KCNQ1* deletion cases have been reported, and the development of a *KCNQ1* deletion model helps improve the treatment of this disease. Even though *KCNQ1*-knockout mice were present and seen to exhibit the Jervell and Lange–Nielsen syndrome, the QT prolongation phenotype was thought to be mediated by extracardial factors because I_Ks_ did not participate in the mouse repolarization process [12]. Therefore, we developed an hESC-CM model without *KCNQ1*.

**Fig. 7:**
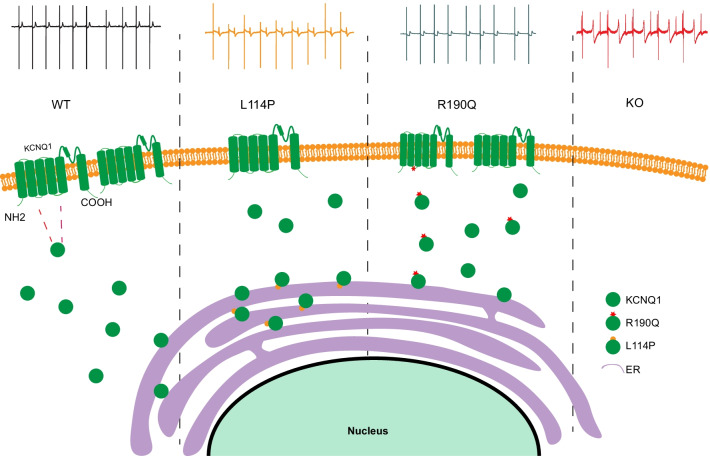
Schematic illustration of KCNQ1 cell membrane expression in four hESC-CMs

In this study, the MEA results showed that the FPD of KCNQ1^−/−^ CMs was significantly prolonged compared with that of WT CMs, indicating that the absence of I_Ks_ prolonged the repolarization time course of CMs. The FPD of KCNQ1^L114P/+^ CMs was prolonged, whereas that of KCNQ1^R190Q/+^ CMs was not different from that of WT CMs. This suggests that the L114P mutation decreases the upper membrane of *KCNQ1*, whereas R190Q does not cause QT prolongation at baseline. Furthermore, KCNQ1^−/−^ CMs showed arrhythmias even at baseline, suggesting that the complete loss of I_Ks_ has a severe effect on myocardial action potentials. When treated with 293B, the FPD of KCNQ1^−/−^ CMs was not significantly prolonged, whereas that of KCNQ1^L114P/+^ and KCNQ1^R190Q/+^ CMs was, indicating that this insensitivity was due to the deletion of Kv7.1 channel encoded by *KCNQ1*. The baseline phenotype initially reflected the success of model construction and the phenotype of the disease. A schematic of the mechanism can be seen in Fig. 7.

LQT1 often occurs during exercise and emotional arousal; therefore, we used ISO to simulate sympathetic excitation. After ISO treatment, WT, KCNQ1^−/−^, and KCNQ1^L114P/+^ CMs showed significant shortening of the FPD, indicating that they responded to ISO’s excitement. However, KCNQ1^R190Q/+^ CMs showed insensitivity to ISO stimulation, confirming that this mutation indeed causes the passivation of Kv7.1 channel to PKA stimulation. Under ISO stimulation, all models showed aggravation of the arrhythmia phenotype, partially simulated LQT1 caused by *KCNQ1* mutation under sympathetic excitation. Subsequently, we used common clinical LQT drug treatments to explore the response of different mutations to different drugs. The use of propranolol significantly prolonged the FPD of the three types of CMs and maintained cardiac rhythm stability in KCNQ1^−/−^, KCNQ1^L114P/+^, and KCNQ1^R190Q/+^ CMs, indicating that β-blockers have good therapeutic effects on LQT with different mechanisms. Amiodarone has a greater prolongation effect on the FPD in the three models; however, amiodarone treatment may result in reduced pulsation of KCNQ1^−/−^ CMs, suggesting that it is not be suitable for treating patients with large fragment *KCNQ1* deletion. MgCl_2_ treatment can reduce the FPD of the three models and has a good effect on heart rhythm stability; this reflects the importance of magnesium supplementation for patients under treatment for LQT.

This study established a hESC-CM model of *KCNQ1* deletion, clarified the relationship between the genotype and phenotype of *KCNQ1*, and bridged the gap in terms of a human model of *KCNQ1* deletion. Moreover, KCNQ1^−/−^, KCNQ1^L114P/+^, and KCNQ1^R190Q/+^ CMs showed different responses to different drug interventions, and their phenotypic changes were consistent with the mechanisms proposed in previous studies, indicating that the artificial absence and pitting myocardial model can reflect the LQT phenotype. As a good disease model, it shows great potential for application in precision treatment and drug screening. However, because the phenotype of hESC-CMs is closer to immature myocardium, there are some differences between hESC-CMs and adult myocardium in electrophysiological phenotype. This difference makes the hESC-CMs model unable to fully simulate the pathological phenotype and drug response of human myocardium. At the same time, we use the 2D myocardial differentiation method in this study, and the complete heart structure is not constructed, which may also lead to the difference between the drug response of this model and that under normal physiological conditions. These problems limit the application of the model in the clinical field. In the follow-up study, we will consider constructing engineered myocardial tissue (EHT) model to better approach the physiological phenotype.

## Conclusion

In this study, we developed a *KCNQ1* defect model using the CRISPR/Cas9 system. Simultaneous electrophysiological detection was performed on KCNQ1^−/−^, KCNQ1^L114P/+^, and KCNQ1^R190Q/+^ CMs. The KCNQ1^−/−^ CM model showed significant QT interval prolongation, arrhythmia, and sensitivity to other ion channel blockers. This model can be used as an important tool to improve our understanding of the basic pathological mechanism of *KCNQ1* dysfunction, define the genotype–phenotype correspondence, and promote drug development. Furthermore, under different intervention conditions, the phenotypes of KCNQ1^L114P/+^ and KCNQ1^R190Q/+^ CMs showed different responses, suggesting that 114 and 190 have different pathogenic mechanisms. It provides an effective model for studying the different pathogenic mechanisms of LQT and confirms the feasibility of preparing a single-gene genetic disease model through gene editing.

## Supplementary Information

**Additional file 1.** Supplementary Figure legends.

**Additional file 2. Supplemental Figure 1.** KCNQ1 point mutation did not affect the pluripotency nature of hESC.

**Additional file 3. Supplemental Figure 2.** KCNQ1 knockout and point mutations(L114P、R190Q)did not affect the ability of cardiac differentiation.

## Data Availability

All data generated or analyzed during this study are included in this published article.

## Abbreviations

IKs: Delayed-rectifier potassium current
hESC: Human embryonic stem cell
LQTS: Long-QT syndrome
MEA: Microelectrode array
CM: Cardiomyocyte
hiPSC-CMs: Human induced pluripotent stem cell-derived CMs
EAD: Early post-depolarization
sgRNA: Single-stranded guide RNA
ISO: Isoproterenol
FPD: Field potential duration
WT: Wild type
