# Supplementary material for: KCNQ1-deficient and KCNQ1-mutant human embryonic stem cell-derived cardiomyocytes for modeling QT prolongation
Source: Stem Cell Res Ther. 2022 Jun 28;13:287. doi: 10.1186/s13287-022-02964-3 (PMC9241307; doi:10.1186/s13287-022-02964-3)
Supplement: Supplementary file 1 — Additional file 1. Supplementary Figure legends. [file 13287_2022_2964_MOESM1_ESM.docx]

**Supplementary Figure legends**

**Supplemental Figure 1. KCNQ1 point mutation did not affect the pluripotency nature of hESC.**

**A.** Immunofluorescence staining showed hESCs colonies for the pluripotency markers SSEA4,OCT4,TRA-1-60 and NANOG. Scale bar=75 μm.

**B.** HE staining demonstrates in vivo teratoma of R190Q hESC cell line.

**C.** RT-PCR showed that the expression of stem cell pluripotency genes OCT4, DPPA4, REX1, NANOG and SOX2 did not change significantly on R190Q hESC cell line.

**D.** R190Q hESC cell line showed normal 46 X, X karyotype.

**E.** Schematic of cardiac differentiation using small molecule-based protocols.

**Supplemental Figure 2. KCNQ1 knockout and point mutations(L114P、R190Q)did not affect the ability of cardiac differentiation.**

1. Schematic diagram of human KCNQ1 knockout sites and L114 and R190Q gene point mutation editing sites.

**B.** Immunofluorescence staining of cardiomyogenic differentiation markers TNNT2 and α-actinin. Scale bar = 25μm.

**C,D.** Representative staining and quantitative analysis of unpurified TNNT2 on day 10 were detected by flow cytometry.

**E.** CCK8 showed the relative activity of the four cardiomyocytes.

**F.** The membrane diagram of western blot experiment in Figure 1A.

Results are presented as means ± S.E.M.( *P < 0.05, **P <0.01, ***P < 0.001, ****P <0.0001)

**Supplementary Tables**

**Supplementary Table 1:** **Primer sequences used for RT-PCR**

| Gene | Forward primers（5′-3′） | Reward primers（5′-3′） |
| --- | --- | --- |
| SOX2 | ACAGATGCAACCGATGCACC | TGGAGTTGTACTGCAGGGCG |
| OCT4 | CCTGAAGCAGAAGAGGATCACC | AAAGCGGCAGATGGTCGTTTGG |
| NANOG | GAATATTTGGAAACAGCTTGGT | CAAAGTCAGTAAAAAGCATAAG |
| DPPA4 | CTCCACAGAGAAGTCGAGGGAA | GGTTGTCAGTGTGCTCTGCCTT |
| REX1 | GCCTTATGTGATGGCTATGTGT | ACCCCTTATGACGCATTCTATGT |

**Supplementary Table 2:** **Antibodies for immunofluorescence, flow cytometry and western blot in this study**

| Antibody | Application | Dilution | Manufacturer | Catalog |
| --- | --- | --- | --- | --- |
| Anti-OCT4 | IF | 1:100 | Santa Cruz | sc-9081 |
| Anti-SSEA4 | IF | 1:100 | Santa Cruz | sc-21706 |
| Anti-TNNT2 | IF  FC | 1:100  1:200 | Santa Cruz | [sc-20025](https://www.scbt.com/zh/p/troponin-t-c-antibody-ct3?requestFrom=search) |
| Anti-KCNQ1 | WB | 1:500 | Santa Cruz | sc-515884 |
| Anti-GAPDH | WB | 1:500 | Abcam | ab181602 |
| Goat anti-Mouse IgG Alexa Fluor 594 | IF | 1:200 | Invitrogen | A21145 |
| Goat anti-Rabbit IgG Alexa Fluor 488 | IF | 1:200 | Invitrogen | A32731 |
| Goat anti-Rabbit IgG (H + L) IRDye 800CW | WB | 1:20000 | LI-COR | 926-32211 |
| Goat anti-Mouse IgG (H + L) IRDye 800CW | WB | 1:20000 | LI-COR | 926-32211 |

IF: Immunofluorescence

FC: Flow cytometry

WB: Western Blot
