# Supplementary figures and images for: KCNQ1-deficient and KCNQ1-mutant human embryonic stem cell-derived cardiomyocytes for modeling QT prolongation
Source: Stem Cell Res Ther. 2022 Jun 28;13:287. doi: 10.1186/s13287-022-02964-3 (PMC9241307; doi:10.1186/s13287-022-02964-3)

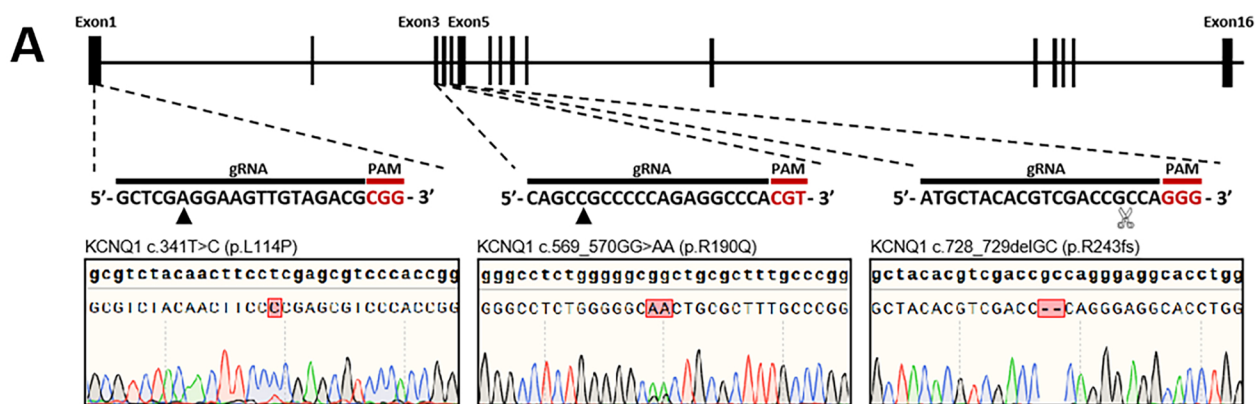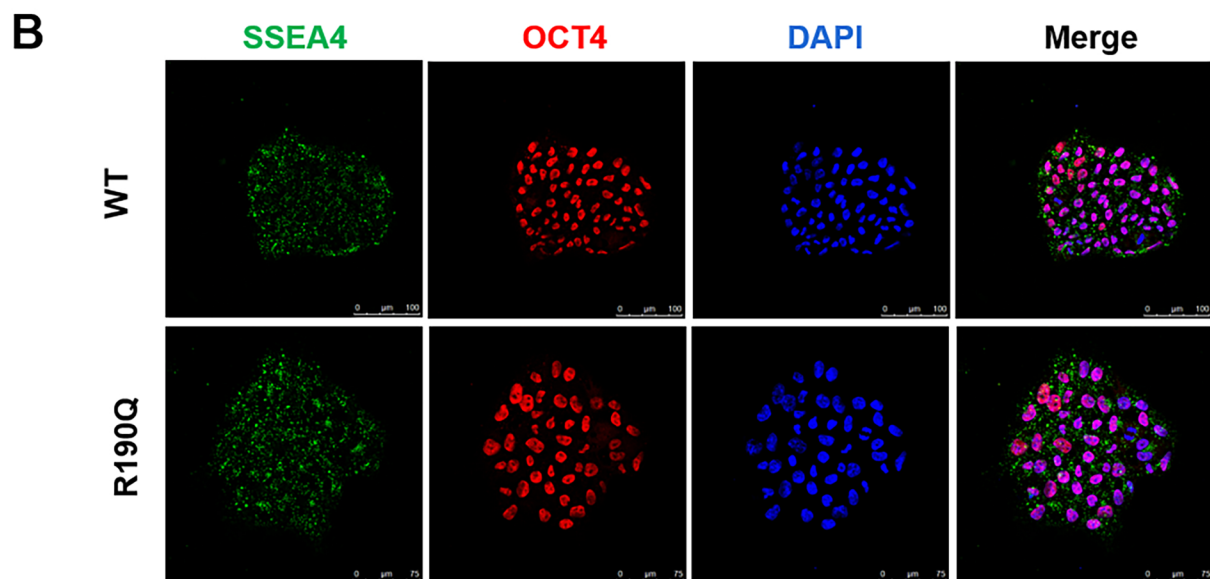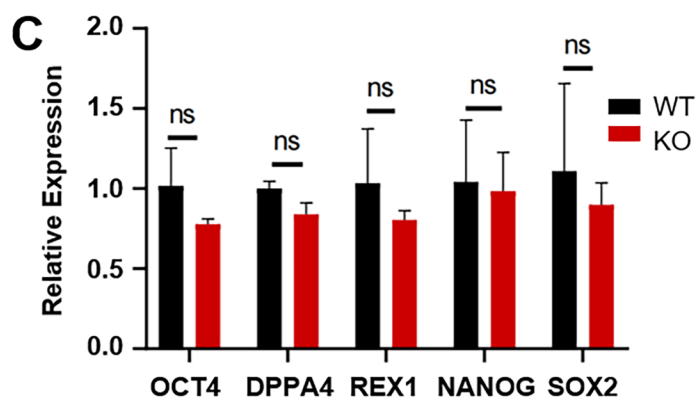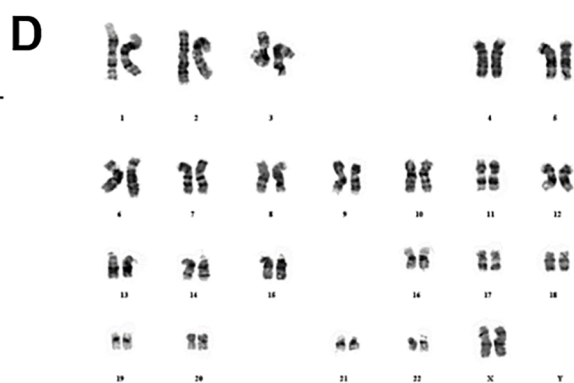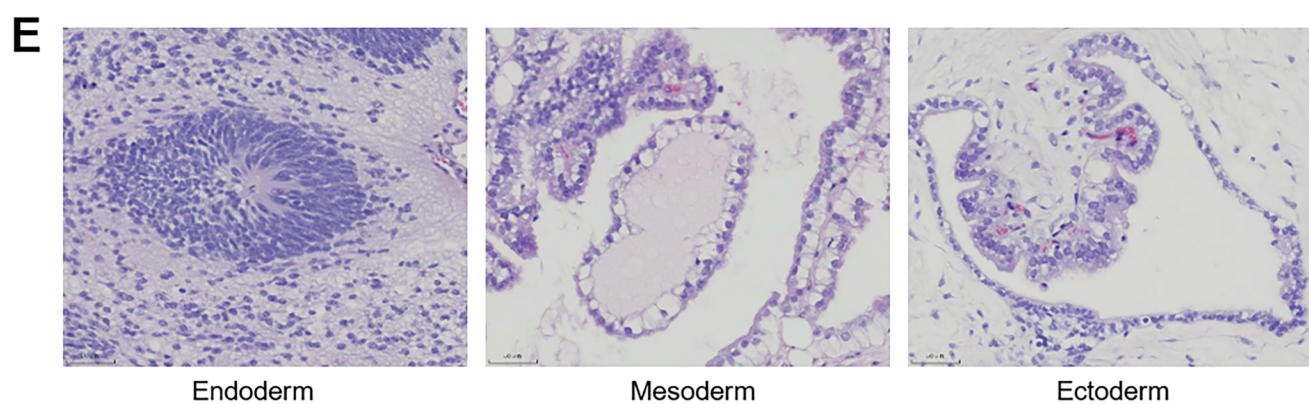

Supplement: Supplementary file 2 — Additional file 2. Supplemental Figure 1. KCNQ1 point mutation did not affect the pluripotency nature of hESC. [file 13287_2022_2964_MOESM2_ESM.pdf]

**A**

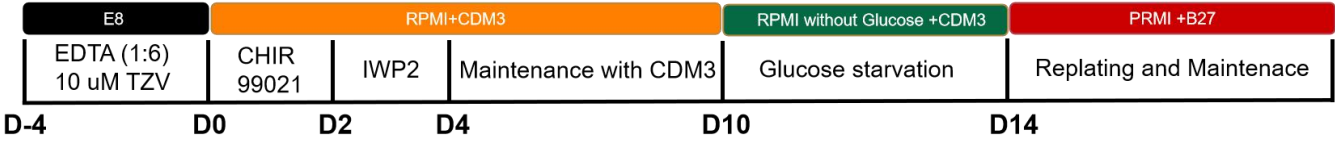

**B**

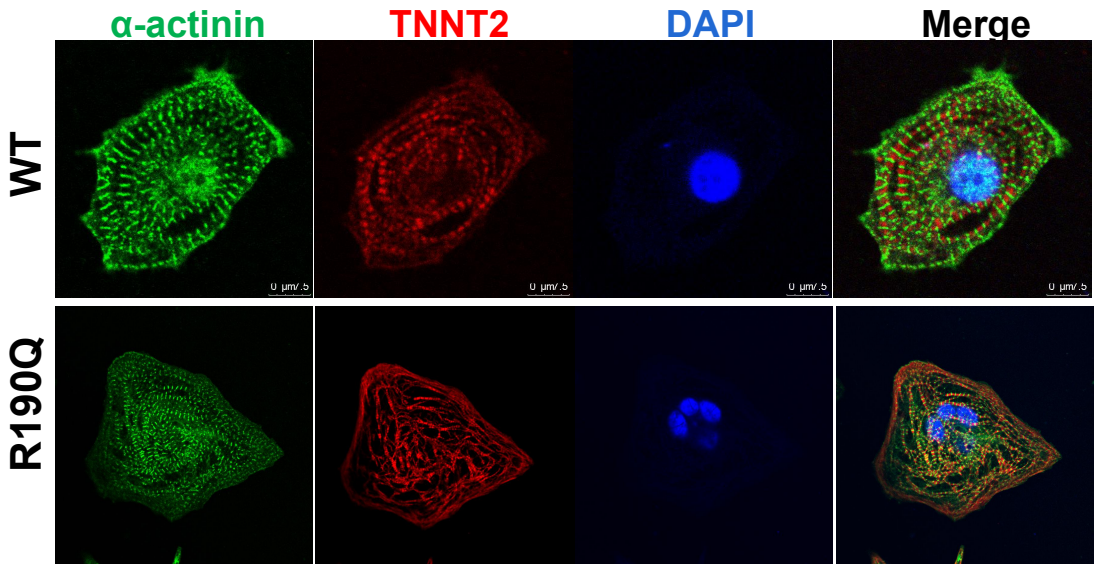

**C**

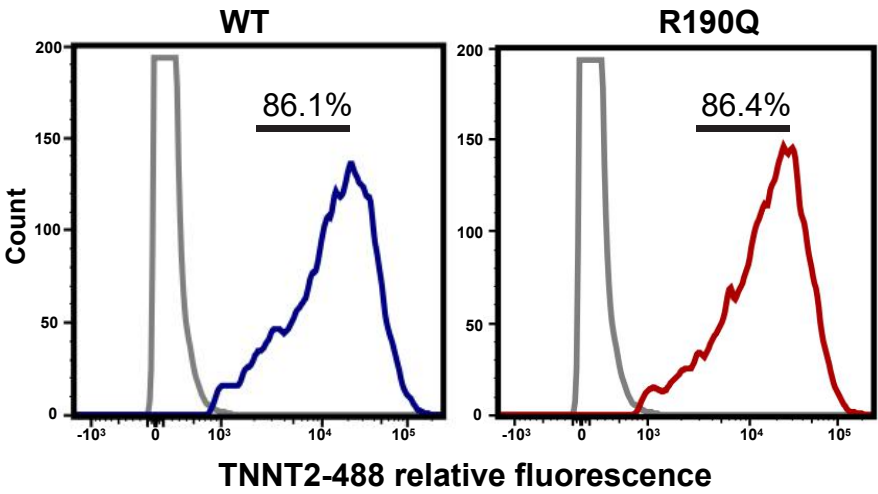

**D**

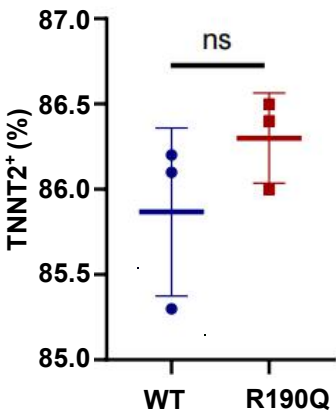

**E**

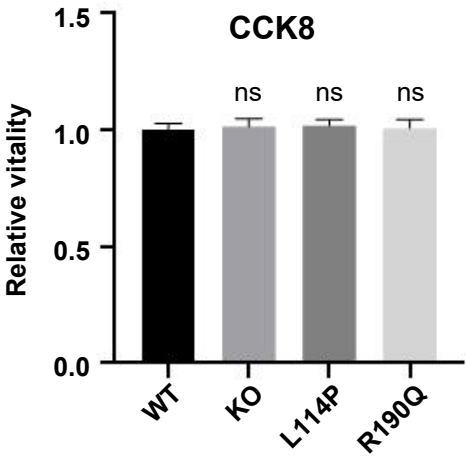

**F**

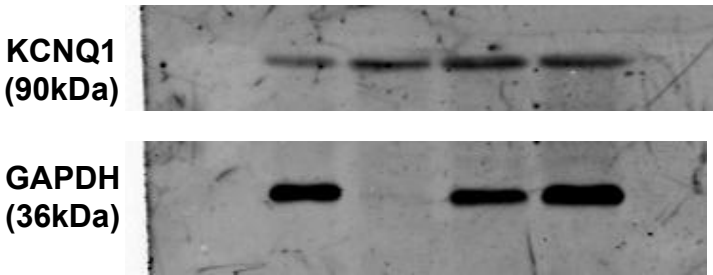

Supplement: Supplementary file 3 — Additional file 3. Supplemental Figure 2. KCNQ1 knockout and point mutations(L114P、R190Q)did not affect the ability of cardiac differentiation. [file 13287_2022_2964_MOESM3_ESM.pdf]
